# Culinary nutrition course equips future physicians to educate patients on a healthy diet: an interventional pilot study

**DOI:** 10.1186/s12909-021-02702-y

**Published:** 2021-05-17

**Authors:** Nathan I. Wood, Rebecca D. Gleit, Diane L. Levine

**Affiliations:** 1 Department of Internal Medicine, Yale New Haven Hospital, 1450 Chapel Street, Private 220, New Haven, CT 06511 USA; 2 Department of Sociology, Stanford University, 450 Jane Stanford Way, Building 120, Room 160, Stanford, CA 94305 USA; 3 Department of Internal Medicine, Wayne State University, 4201 St. Antoine Boulevard, Detroit, MI 48201 USA

**Keywords:** Cooking, Diet, Food, Nutrition, Medical education, Curriculum

## Abstract

**Background:**

Poor-quality diet is associated with one in five deaths globally. In the United States, it is the leading cause of death, representing a bigger risk factor than even smoking. For many, education on a healthy diet comes from their physician. However, as few as 25% of medical schools currently offer a dedicated nutrition course. We hypothesized that an active learning, culinary nutrition experience for medical students would improve the quality of their diets and better equip them to counsel future patients on food and nutrition.

**Methods:**

This was a prospective, interventional, uncontrolled, non-randomized, pilot study. Ten first-year medical students at the Wayne State University School of Medicine completed a 4-part, 8-h course in culinary-nutritional instruction and hands-on cooking. Online assessment surveys were completed immediately prior to, immediately following, and 2 months after the intervention. There was a 100% retention rate and 98.8% item-completion rate on the questionnaires. The primary outcome was changes in attitudes regarding counselling patients on a healthy diet. Secondary outcomes included changes in dietary habits and acquisition of culinary knowledge. Average within-person change between timepoints was determined using ordinary least squares fixed-effect models. Statistical significance was defined as *P* ≤ .05.

**Results:**

Participants felt better prepared to counsel patients on a healthy diet immediately post-intervention (coefficient = 2.8; 95% confidence interval: 1.6 to 4.0 points; *P* < .001) and 2 months later (2.2 [1.0, 3.4]; *P* = .002). Scores on the objective test of culinary knowledge increased immediately after (3.6 [2.4, 4.9]; *P* < .001) and 2 months after (1.6 [0.4, 2.9]; *P* = .01) the intervention. Two months post-intervention, participants reported that a higher percentage of their meals were homemade compared to pre-intervention (13.7 [2.1, 25.3]; *P* = .02).

**Conclusions:**

An experiential culinary nutrition course may improve medical students’ readiness to provide dietary counselling. Further research will be necessary to determine what effects such interventions may have on the quality of participants’ own diets.

**Supplementary Information:**

The online version contains supplementary material available at 10.1186/s12909-021-02702-y.

## Background

Globally, an unhealthy diet was responsible for 11 million deaths in 2017 [1]. In the United States, poor-quality diet is the leading cause of death [2], representing a bigger risk factor for morbidity and mortality than obesity, hypertension, hypercholesterolemia, physical inactivity, and even smoking [3]. While positive dietary changes represent an obvious solution to decreasing morbidity and mortality, many patients are still unsure of what changes to make and/or how to enact them. As the quality of an individual’s diet is directly correlated with their nutritional knowledge [4–10], a lack of this knowledge, therefore, represents a major obstacle for many patients looking to adopt a healthy diet [11–13].

Physicians are both trusted and influential sources of nutritional information for patients seeking to improve their diet. Nearly 80% of patients who seek dietary information from their doctors make a subsequent change in their eating habits [14]. For this reason, a crucial element of the World Health Organisation’s United Nations Decade of Action on Nutrition 2016–2025 involves doctors supporting and advocating for evidence-based nutritional practices [15]. Doctors do recognise this important role they have as an educational resource, with as many as 95% of surveyed physicians reporting that they believe it’s their personal responsibility to provide nutrition counselling to their patients [16]. But this belief has yet to adequately translate into clinical practice [2], with nutrition education being provided in as few as 12% of office visits [17].

A likely cause of this discrepancy is physicians’ perceived lack of preparedness to effectively counsel patients on diet. Fewer than one in six physicians feels highly confident in their ability to discuss nutrition with patients [18, 19]. Medical students and doctors who most routinely provide counselling are those who practice a healthy diet themselves [20–24], suggesting that doctors’ own knowledge of food and nutrition may play a key role in patient education.

Physicians, however, report that their formal training received in nutrition and diet counselling, particularly in medical school, is inadequate [25–29]. In fact, only 25% of medical schools provide a dedicated nutrition course, with this coursework frequently being done via online modules [30]. On average, medical schools in the United States provide only 19 h of nutrition education – six fewer hours than the minimum 25 recommended by the National Academy of Medicine. In all, 71% of medical schools – serving 75% of US medical students – fail to provide their students with the minimum recommended nutrition education during their 4 years of training [31]. Outside of the United States, education for medical students has similarly and repeatedly been shown to be insufficient in enabling future physicians to confidently provide nutrition counselling for their patients [29].

The purpose of this study was to evaluate the efficacy of a hands-on culinary nutrition curriculum in influencing first-year medical students’ personal dietary habits and perceived preparedness to counsel patients on a healthy diet. Educational interventions aimed at addressing doctors’ nutritional knowledge gaps are becoming increasingly common in the medical education and healthcare landscapes. The most successful nutrition education interventions, recent literature has found, are practical and emphasize skill development instead of mere knowledge acquisition [32, 33]. This finding is congruent with recent pedagogical research that has demonstrated the superiority of active learning in engagement and content mastery compared to lecturing alone, particularly in the science, technology, engineering, and mathematics (STEM) fields [34–36]. For this reason, we engaged with the burgeoning trend of active learning instruction in undergraduate medical education [37] to design this hands-on curriculum. We hypothesized that an active learning intervention would improve the quality of participants’ diets and better equip them to counsel their future patients on food and nutrition.

## Methods

### Study design and sample

The investigation was a single-center, prospective, interventional, uncontrolled, non-randomised, pilot study. All first-year medical students at the Wayne State University School of Medicine (WSUSOM) in Detroit, Michigan who completed the required Clinical Nutrition course, approximately 300, were eligible to participate and were invited via listserv emails in the 8 weeks leading up to the intervention. All interested students who could commit to attending the course in full then participated in and completed the intervention. The potential benefits of expanding the assessment of this intervention with a controlled trial are discussed further in the Discussion. The study was approved by the Wayne State University Institutional Review Board (IRB) under exempt review. All participants were older than 18 years of age and able to provide informed consent, although the need for written informed consent was waived per the IRB. In the interest of maximizing participation by eliminating the request to divulge potentially sensitive information, no demographics were collected from participants as part of this pilot study.

### Intervention

Participants completed a four-session, eight-hour intervention called “Culinary Nutrition: A Practical Course.” The course was held at the Wayne State University Food Sciences Laboratory over four consecutive evenings in May 2018, approximately 1 month after all traditional first-year medical students at WSUSOM completed their required 40-h Clinical Nutrition course. The intervention’s curriculum was designed as a practical complement to the lecture-based Clinical Nutrition course. Each of the 4 weeks of the Clinical Nutrition course had its own theme: (1) micronutrients, (2) obesity, (3) diabetes, and (4) cardiovascular disease. Correspondingly, each of the four sessions of the Culinary Nutrition course was thematically congruent with one of these four broad themes addressed in the traditional Clinical Nutrition course.

**Fig. 1:**
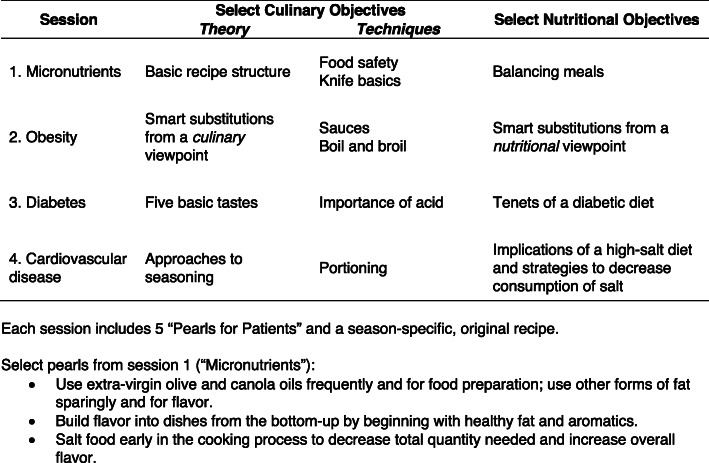
Structure of the “Culinary Nutrition: A Practical Course” Intervention Curriculum. The intervention course, “Culinary Nutrition: A Practical Course,” included four sessions, each complete with both culinary and nutrition objectives and major takeaways distilled into five “Pearls for Patients”

During each of the four two-hour sessions, participants received approximately 20 min of culinary theory didactic instruction, 10 min of demonstrated culinary technique instruction, 80 min of supervised cooking in small groups, and 10 min of an interactive nutrition discussion. For instance, in week 2, students learned about the five French mother sauces, were shown how to use safe knife skills to cube a butternut squash, together made a healthier version of mac and cheese (featuring one of the five French mother sauces as well as whole-wheat pasta and extra vegetables), and discussed the importance of whole grains and fiber for glycemic control. Figure 1 further describes the structure of the intervention.

The course curriculum was developed and taught by N.I. Wood, a rising fourth year WSUSOM medical student and professional culinary arts student at the time of the intervention. Wood completed the WSUSOM Clinical Nutrition course himself in 2015. The curriculum was reviewed by the course director for the required Clinical Nutrition course, T. Reinhard, a Registered Dietician and Fellow of the Academy of Nutrition and Dietetics. The total cost of the course was approximately $500.

### Measures and procedures

Participants completed survey questionnaires at three timepoints: immediately pre-intervention (time 1), immediately post-intervention (time 2), and 2 months post-intervention (time 3). The main exposure of the study – participation in the culinary nutrition course – occurred between survey waves 1 and 2. The surveys were anonymous, completed online using SurveyMonkey.com (SurveyMonkey, San Mateo, California), and took an estimated 10 min to complete at each timepoint. The questionnaires were informed by the literature and developed in an iterative process to measure the impact of the curriculum delivered. They were then reviewed by a content expert in survey methodology. No standard or validated questionnaires for assessing the efficacy of culinary nutrition curricula were available at the time of study conception. The surveys asked participants to quantitatively rate their behaviours and attitudes regarding health, wellness, and anticipated effectiveness in counselling patients about a healthy diet on a Likert scale from 0 (“do not agree at all”) to 10 (“completely agree”) (see supplementary data for questionnaires). Each questionnaire also included an objective test of participants’ culinary knowledge. Anonymous codenames generated by participants were used to link individuals’ responses across the three survey waves.

The primary outcomes were within-subject changes in medical students’ attitudes about counselling patients on the tenets of a healthy diet. Specifically, participants were asked to rate how prepared, motivated, and excited they were to counsel patients on practicing a healthy diet. Secondary outcomes included changes in subjects’ culinary knowledge over time and whether they reported positive changes in personal dietary habits between the pre- and post-intervention timepoints, such as eating more homemade and less pre-prepared food.

### Data analysis

All analyses were conducted in Stata 14.2 (StataCorp, College Station, Texas). After calculating group means for each outcome variable at each of the three timepoints, we used ordinary least-squares (OLS) fixed-effect (FE) models to estimate the average within-person change in each outcome between timepoints. OLS was used because all outcomes were continuous. With the exception of self-reported “percent of meals homemade,” all were measured from 0 to 10. A score of 5 was considered neutral. A score of less than 5 was considered negative, and a score of greater than 5 was considered positive. Scores of 7 and higher were considered “highly positive,” and scores of 3 and lower were considered “highly negative.” For all but four models, there were no missing data for any individual-time observation. In those four models, one timepoint had only nine valid responses; for these, the missing observation was deleted listwise, and the models included 29 rather than 30 observations.

The main predictor variable was time, which was included in the models as a three-category factor variable, with baseline (time 1) as the reference group. The models included this time variable and individual fixed effects. Individual fixed effects allowed us to account for the differing starting positions of each participant at baseline. Moreover, FE models estimate standard errors based on within-person change over time, which nets out any potential confounders due to stable differences across individuals – such as demographics, stable dietary restrictions, etc. – from our analyses. As such, our estimates for the impact of the intervention can be interpreted as causal with the large assumption that nothing else systematically changed at the same times to also affect the outcome variables. Given the relatively small size of this pilot study, we did not test for any effect modifiers. For all analyses, statistical significance is defined as *P* ≤ .05.

## Results

All first-year medical students, approximately 300, were eligible. Ten students volunteered to participate, were examined for eligibility, were confirmed eligible, and were then included in the pilot study. There was a 100% retention rate; every participant attended each of the four sessions. There was a 98.8% survey item completion rate for the associated three waves of questionnaires.

### Attitudes about Counselling patients on healthy lifestyle

At baseline, the participants reported being both highly motivated (mean = 8.2 points) and excited (mean = 8.2 points) to counsel patients on practicing a healthy lifestyle. In contrast, participants at baseline did not rate themselves as feeling highly prepared (mean = 4.8 points) to do so (Table 1). On average, respondents’ self-reported preparedness was significantly higher immediately post-intervention (coefficient = 2.8 points; 95% confidence interval [CI]: 1.6 to 4.0 points; *P* < .001) and 2 months post-intervention (2.2 [1.0, 3.4]; *P* = .002) compared to baseline. There was no significant decline in respondents’ preparedness between the immediately post- and 2 months post-intervention surveys (− 0.6 [− 1.8, 0.6]; *P* = .32) (Table 2). Neither self-reported motivation nor self-reported excitement changed significantly from baseline at either of the follow-up timepoints (Table 2).

**Table 1:** Self-Rated Group Mean Scores from Participants at Pre-, Immediately Post-, and 2 Months Post-Intervention

	Time 1 Pre-Intervention		Time 2 Immediately Post-Intervention		Time 3 2 Months Post-Intervention	
	Mean	n	Mean	n	Mean	n
**Attitudes about Counseling Patients**						
I am [x] to effectively counsel patients on how to practice a healthy lifestyle						
Motivated	8.2	10	9.0	10	8.3	10
Excited	8.2	10	8.9	10	8.7	9
Prepared	4.8	10	7.6	10	7.0	10
I have the [x] knowledge necessary to effectively counsel patients on how to practice a healthy lifestyle						
Medical	6.0	10	7.9	10	7.2	10
Nutritional	5.9	10	7.7	10	7.2	10
Culinary	4.5	10	7.5	10	7.1	10
**Objective Culinary Knowledge**^a^						
Total score	5.3	9	8.8	10	6.9	10
**Attitudes about Own Lifestyle**						
I have the [x] necessary to practice a healthy lifestyle						
Motivation	7.8	10	8.6	10	8.0	10
Medical knowledge	6.9	10	8.0	10	7.3	10
Nutritional knowledge	6.4	10	7.7	10	7.7	10
Culinary theory/knowledge	4.7	10	7.4	10	6.9	10
Culinary technique/skills	4.5	10	7.6	10	7.5	10
I can use culinary knowledge and skills to positively impact my [x].						
Health	8.8	9	9.0	10	8.9	10
Wellness	8.8	10	8.9	10	9.0	10
**Self-Reported Behaviors**						
Estimated number of times *per week* you eat the following types of meals						
Restaurants	3.2	10	2.9	10	2.9	10
Pre-prepared	2.3	10	2.3	9	1.0	10
Homemade	14.7	10	14.5	10	17.8	10
Percent of meals homemade	64.4%	10	68.5%	10	78.1%	10

^a^Objective culinary knowledge is the total score (0–10) from a 10-question multiple choice assessment, with 1 point given to each correct answer

**Table 2:** Average Within-Subject Change in Participants’ Self-Rated Scores Between Pre- and Post-Intervention Timepoints

	Time 1 ➔ Time 2		Time 1 ➔ Time 3		n
	Estimate	95% CI	Estimate	95% CI	
**Attitudes About Counseling Patients**					
I am [x] to effectively counsel patients on how to practice a healthy lifestyle					
Motivated	0.8	(−0.5, 2.1)	0.1	(−1.2, 1.4)	30
Excited	0.7	(−0.4, 1.8)	0.63	(−0.5, 1.8)	29
Prepared	2.8***	(1.6, 4.0)	2.2**	(1.0, 3.4)	30
I have the [x] knowledge necessary to effectively counsel patients on how to practice a healthy lifestyle.					
Medical	1.9**	(0.7, 3.1)	1.2	(−0.003, 2.4)	30
Nutritional	1.8***	(1.0, 2.6)	1.3**	(0.5, 2.1)	30
Culinary	3.0***	(1.8, 4.2)	2.6***	(1.4, 3.8)	30
**Objective Culinary Knowledge**^a^					
Total score^b^	3.6***	(2.4, 4.9)	1.6*	(0.4, 2.9)	29
**Attitudes about Own Lifestyle**					
I have the [x] necessary to practice a healthy lifestyle					
Motivation	0.8	(−0.2, 1.8)	0.2	(−0.8, 1.2)	30
Medical knowledge	1.1*	(0.2, 2.0)	0.4	(−0.5, 1.3)	30
Nutritional knowledge	1.3*	(0.2, 2.4)	1.3*	(0.2, 2.4)	30
Culinary theory/knowledge	2.7***	(1.6, 3.8)	2.2**	(1.1, 3.3)	30
Culinary technique/skills	3.1***	(1.9, 4.3)	3.0***	(1.8, 4.2)	30
I can use culinary knowledge and skills to positively impact my [x].					
Health	0.3	(−0.9, 1.5)	0.2	(−1.0, 1.4)	29
Wellness	0.1	(−1.0, 1.2)	0.2	(−0.9, 1.3)	30
**Self-Reported Behaviors**					
Estimated number of times *per week* you eat the following types of meals					
Restaurants	−0.3	(−1.1, 0.5)	− 0.3	(− 1.1, 0.5)	30
Pre-prepared	−0.2	(−1.6, 1.3)	−1.3	(−2.7, 0.1)	29
Homemade^b^	−0.2	(−3.1, 2.7)	3.1*	(0.2, 6.0)	30
Percent of meals homemade	4.1	(−7.5, 15.7)	13.7*	(2.1, 25.3)	30

*n* the number of person-time observations in each model, *CI* Confidence interval

*P < .05 **P < .01 ***P < .001

Estimates and confidence intervals obtained from linear regression models with individual fixed effects

^a^Objective culinary knowledge is the total score from a 10-question multiple choice assessment, with 1 point given to each correct answer

^b^For these variables (total score, homemade), there was a significant within-subject change between time 2 and time 3 at the *P* < .05 level. For all other variables, there was no significant within-subject change between time 2 and time 3

**Fig. 2:**
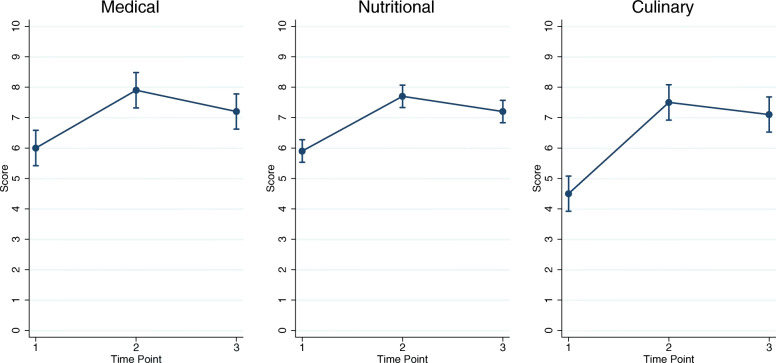
Participants’ Self-Reported Mastery of Necessary Medical, Nutritional, and Culinary Knowledge to Counsel Patients. Medical students’ self-ratings of whether they have the medical, nutritional, and culinary knowledge to effectively counsel patients on a healthy lifestyle increased significantly from pre-intervention (time 1) to immediately post-intervention (time 2). Gains were sustained two months post-intervention (time 3). 83% confidence intervals obtained from linear regression models with individual fixed effects are shown

Participants also rated the effectiveness of the training that they had received in preparing them to counsel patients on a healthy lifestyle. Specifically, the questionnaires asked if they felt they had the medical, nutritional, and culinary knowledge necessary to counsel patients on a healthy lifestyle. At baseline, participants on average felt that they had the medical knowledge (mean = 6.0 points) and nutritional knowledge (mean = 5.9 points) necessary. They did not feel that they had the necessary culinary knowledge, however (mean = 4.5 points) (Fig. 2). Immediately post-intervention, there were statistically significant increases in participants’ confidence in their medical (1.9 [0.7, 3.1]; *P* = .004), nutritional (1.8 [1.0, 2.6]; *P* < .001), and culinary (3.0 [1.8, 4.2]; *P* < .001) knowledge compared to baseline. There were no significant declines at 2 months post-intervention compared to immediately post-intervention in medical (− 0.7 [− 1.9, 0.5]; *P* = 0.24), nutritional (− 0.5 [− 1.3, 0.3]; *P* = .19) or culinary (− 0.4 [− 1.6, 0.]; *P* = .49) knowledge.

### Additional findings

Participants reported at baseline that they believed that culinary knowledge could be used to positively impact both their health (mean = 8.8 points) and wellness (mean = 8.8 points) (Table 1). There were no significant changes in participants’ belief in the possible impact of culinary knowledge on health from baseline when surveyed immediately post-intervention (0.3 [− 0.9, 1.5]; *P* = .64) and 2 months post-intervention (0.2 [− 1.0, 1.4]; *P* = .77). The same was found for their belief about culinary knowledge impacting wellness; there were no significant changes between baseline and either the immediately post-intervention follow-up (0.1 [− 1.0, 1.2]; *P* = .85) or the 2 months post-intervention follow-up (0.2 [− 0.9, 1.3]; *P* = .70).

**Fig. 3:**
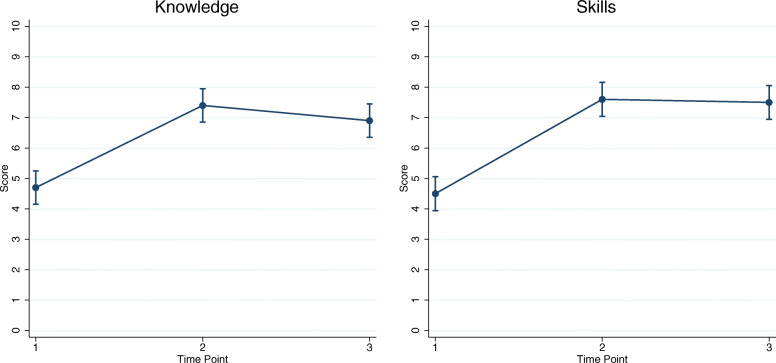
Participants’ Self-Reported Mastery of Necessary Culinary Knowledge and Skills to Practice a Healthy Lifestyle. Medical students’ self-ratings of whether they have the culinary knowledge and skills to practice a healthy lifestyle increased significantly from pre-intervention (time 1) to immediately post-intervention (time 2). Gains were sustained two months post-intervention (time 3). 83% confidence intervals obtained from linear regression models with individual fixed effects are shown

Despite their belief in the importance of culinary knowledge and skills for health and wellness, participants did not initially believe that they had the necessary culinary knowledge (mean = 4.7 points) or skills (mean = 4.5 points) to practice a healthy lifestyle themselves. Post-intervention, the participants felt significantly better equipped (Fig. 3). Mean rating of belief in their culinary knowledge increased to 7.4 points immediately post-intervention (2.7 [1.6, 3.8]; *P* < .001), and mean rating of belief in their culinary skills increased to 7.6 points immediately post-intervention (3.1 [1.9, 4.3]; *P* < .001). Participants’ perceived increase in the adequacy of their training was maintained over time. At 2 months post-intervention, there were no significant declines in self-rated culinary knowledge (− 0.5 [− 1.6, 0.6]; *P* = .37) or skills (− 0.1 [− 1.3, 1.1]; *P* = .86) compared to immediately post-intervention.

**Fig. 4:**
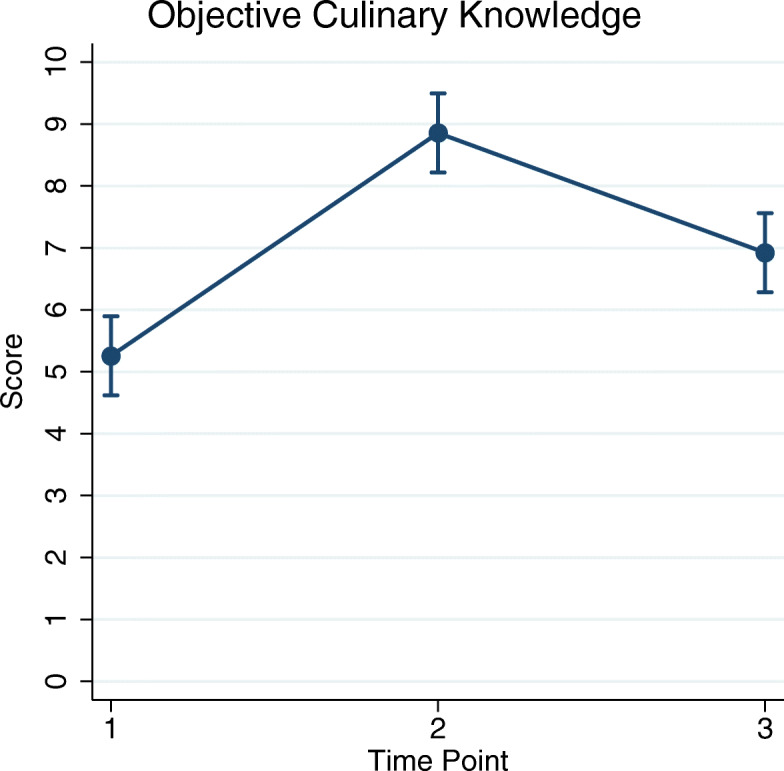
Participants’ Scores on an Assessment of Objective Culinary Knowledge at Pre- and Post-Intervention Timepoints. Medical students’ objective culinary knowledge increased significantly from pre-intervention (time 1) to immediately post-intervention (time 2). Objective culinary knowledge remained significantly higher than baseline at two months post-intervention (time 3). 83% confidence intervals obtained from linear regression models with individual fixed effects are shown

In addition to self-reporting their perceived level of culinary knowledge, participants’ culinary knowledge was also measured via a 10-point objective assessment. Pre-intervention, participants had a mean score of 5.3 points out of a possible 10.0 points. Immediately post-intervention, the mean score had increased significantly to 8.8 points (3.6 [2.4, 4.9]; *P* < .001). By 2 months post-intervention, the mean score had decreased to 6.9 points (Fig. 4). Participants’ objective culinary knowledge scores at 2 months post-intervention were significantly decreased compared to immediately post-intervention (− 2.0 [− 3.2, − 0.8]; *P* = .003) but were still statistically significantly higher than their baseline scores (1.6 [0.4, 2.9]; *P* = .01).

Lastly, surveys also included questions regarding participants’ eating habits and personal attitudes about living a healthy lifestyle. Participants were highly motivated at baseline to practice a healthy lifestyle (mean = 7.8 points) (Table 1); there was no significant change in motivation at either the immediately post-intervention (0.8 [− 0.2, 1.8]; *P* = .10) or 2 months post-intervention (0.2 [− 0.8, 1.2]; *P* = .67) timepoints. Two months post-intervention, participants reported that a significantly higher percentage of their meals were homemade compared to baseline (13.7 [2.1, 25.3]; *P* = .02) (Table 2).

## Discussion

According to the International Food Information Council Foundation’s 2018 Food and Health Survey, the vast majority of patients (78%) who seek dietary information from their physicians change their eating habits as a result of these conversations [14]. Doctors should therefore be familiar with evidence-based nutritional recommendations and educate their patients accordingly. Yet, few physicians feel sufficiently prepared to counsel patients about their diet [18, 19]. A major reason for this is that dedicated nutrition training in medical school is both limited in scope and impractical [31]; it is often virtual lecture-based and thus detached from the real-life skills necessary to prepare nutritious meals and counsel patients [30]. Moreover, even when physicians are educated in nutrition, as they are at the medical school serving as the site of this study, there still frequently exists a knowledge gap in how to apply that knowledge to provide counsel on a healthy diet [29]. To fill these gaps, we tested an interactive, practical, skills-based intervention for medical students designed to improve their knowledge of and confidence with nutrition basics and culinary skills. The ultimate goal of this intervention was to better prepare future physicians to effectively counsel their patients on food and nutrition.

Similar to the findings of Hicks and Murano [18] and Vetter et al. [19], we found that our medical student participants did not feel highly prepared to effectively counsel patients on how to practice a healthy lifestyle pre-intervention: no respondents rated themselves a 7 out of 10 or higher when asked to self-assess their preparation in the baseline survey. However, after the intervention, participants’ self-rated preparedness to counsel patients on a healthy lifestyle was significantly higher. Ninety percent of respondents rated themselves to be a 7 out of 10 or higher on this item in both the immediately post-intervention and 2 months post-intervention surveys, which also reveals the durability of the active learning course’s effects. There were simultaneous increases in participants’ perceptions that they had the medical, nutritional, and culinary knowledge necessary to effectively counsel patients.

Participants’ perception of increased knowledge was mirrored in tests of their objective culinary knowledge, which also increased post-intervention compared to pre-intervention. Despite a decline in objective culinary knowledge at 2 months post-intervention compared to immediately post-intervention, participants’ objective culinary knowledge 2 months post-intervention was still higher overall than before they took the course. We believe that equipping students with this culinary knowledge could reinforce prior learning and lead to a greater sense of mastery and accomplishment in the kitchen, which could then serve to break down one more barrier to their providing practical dietary advice in the hospital or clinic.

In summary, we show that an interactive culinary nutrition course for medical students can improve their culinary knowledge and their confidence in counselling patients about food and nutrition. We find evidence that these improvements can be retained over time, even after a relatively small-scale (8-h), short-term intervention such as this. We attribute the success of this intervention in large part to its practical and interactive nature, which the literature also finds to be the most effective method of nutrition education [32, 33].

Our study has a number of limitations. Primarily, we ran a small, non-randomised, uncontrolled intervention. For a pilot demonstration study such as ours, a convenience sample of students responding to the call for participant volunteers was utilized. This resulted in 10 subjects. Although statistical analyses were done specifically to assess within-person change, replication of this intervention with a larger sample size would afford greater statistical power and further confirmation of this study’s results. A controlled study with randomised assignment to the intervention should also be established to remove self-selection bias. Recall bias and social desirability bias may also have impacted the results. A larger bank of culinary knowledge test questions should be developed and randomised to participants at each of the timepoints to minimise the potential that recall bias contributes to the score increase observed between the objective pre- and post-intervention assessments. The lack of availability of a validated questionnaire for assessing the efficacy of a culinary nutrition curriculum at the time of this study’s conception is also a limitation. Finally, although the surveys were fully anonymous, participant self-reporting may over-report learning and/or under-report remaining doubts if participants felt the desire to “pay back” the instructor and principal investigator, N.I. Wood, with such reviews. Of note, this limitation is somewhat mitigated by the objective assessment of culinary knowledge included at every survey timepoint.

### Implications for future research and practice

Practical culinary nutrition interventions can build on the curriculum used here in a number of ways. Delivering this curriculum to an entire medical school class will be challenging. However, amid the growing landscape of remote learning and video conference calls brought on by the coronavirus disease 2019 (COVID-19) pandemic, we are confident that online or hybrid versions of this course could be piloted as an efficient means of scaling up the curriculum. We are optimistic that the results of this study would be generalisable across these potential new contexts as long as participants continued to cook along at home.

What is most important is to see the impact of the curriculum and hands-on experience on the counselling behaviour of medical students. Therefore, future research should assess the impact of this intervention on the frequency and/or quality of nutrition counselling provided. Such efforts should be paired with ongoing research to further refine the pedagogical approaches that best prepare physicians to help their patients follow a healthy diet. Further research will also be necessary to determine what effect, if any, a practical culinary nutrition course for physician trainees has on the overall healthiness of participants’ diets.

## Conclusions

We conclude that participating in a hands-on culinary nutrition curriculum is an effective method for increasing medical students’ readiness to counsel patients on a healthy diet. We hypothesize that this improvement is due to the intervention’s focus on active learning. Providing nutrition education programs to medical students with hands-on learning opportunities allows them to put into practice the clinical nutrition knowledge learned in the classroom. This promotes the reinforcement of clinical nutrition knowledge, increasing the likelihood that the knowledge is maintained and then can be passed on to patients. It also dismantles the common perception among clinicians that they don’t have the experience or confidence necessary to counsel patients on nutrition. These positive impacts of a practical culinary nutrition course have the potential to bridge the gap between merely acquiring nutrition knowledge and actually implementing routine nutrition education into patient care. To this end, more medical schools should consider incorporating practical culinary nutrition education into their standard curricula.

## Supplementary Information

**Additional file 1.** Questionnaires. Questionnaires completed by participants at three timepoints: (1) pre-intervention, (2) immediately post-intervention, and (3) two months post-intervention.

## Data Availability

The datasets used and/or analysed during the current study are available from the corresponding author on reasonable request.

## Abbreviations

STEM: Science, technology, engineering, and mathematics
WSUSOM: Wayne State University School of Medicine
IRB: Institutional Review Board
OLS: Ordinary least-squares
FE: Fixed-effect
CI: Confidence interval
COVID-19: Coronavirus disease 2019
