# Supplementary material for: Culinary nutrition course equips future physicians to educate patients on a healthy diet: an interventional pilot study
Source: BMC Med Educ. 2021 May 17;21:280. doi: 10.1186/s12909-021-02702-y (PMC8127510; doi:10.1186/s12909-021-02702-y)
Supplement: Supplementary file 1 — Additional file 1. Questionnaires. Questionnaires completed by participants at three timepoints: (1) pre-intervention, (2) immediately post-intervention, and (3) two months post-intervention. [file 12909_2021_2702_MOESM1_ESM.docx]

**TIME 1: PRE-INTERVENTION SURVEY** (30 QUESTIONS) — completed via SurveyMonkey link. Phrases in bold or italics were formatted as such in SurveyMonkey. Each section was shown on a different page of the survey; participants did not see the title for any of the parts. For questions with correct answers, those correct answers are indicated in ***bold italics***. Note that for questions 6 and 7 in Part 3, each correct response was worth 0.2 points. For instance, if a participant named 2 of the 5 basic tastes correctly, they received a score of 0.4 for question 7.

[PART 1: ATTITUDES]

Please rate your agreement with each of the following statements from *0-10*, where

*0* = “do not agree at all,” and

*10* = “completely agree”:

1. I have the **medical** **knowledge** necessary to practice a healthy lifestyle.

0 1 2 3 4 5 6 7 8 9 10

1. I have the **nutritional** **knowledge** necessary to practice a healthy lifestyle.

0 1 2 3 4 5 6 7 8 9 10

1. I have the **culinary theory/knowledge** necessary to practice a healthy lifestyle.

0 1 2 3 4 5 6 7 8 9 10

1. I have the **culinary technique/skills** necessary to practice a healthy lifestyle.

0 1 2 3 4 5 6 7 8 9 10

1. I have the **motivation** necessary to practice a healthy lifestyle.

0 1 2 3 4 5 6 7 8 9 10

1. I can use culinary knowledge and skills to positively impact my **health**.

0 1 2 3 4 5 6 7 8 9 10

1. I can use culinary knowledge and skills to positively impact my **wellness**.

0 1 2 3 4 5 6 7 8 9 10

[PART 2: BEHAVIOR]

Please estimate the number of times *per week* you engage in the following behaviors:

1. Eat meals from a **restaurant** (dine-in, delivery, and takeout).

0 1 2 3 4 5 6 7 8 9 10+

1. Eat **pre-prepared meals** NOT from a restaurant (e.g., supermarket deli, frozen, or microwavable meals, etc.).

0 1 2 3 4 5 6 7 8 9 10+

1. Make a homemade **breakfast**.

0 1 2 3 4 5 6 7 8 9 10+

1. Make a homemade **lunch**.

0 1 2 3 4 5 6 7 8 9 10+

1. Make a homemade **dinner**.

0 1 2 3 4 5 6 7 8 9 10+

1. Eat **leftovers**/previously prepared homemade meals.

0 1 2 3 4 5 6 7 8 9 10+

1. For whatever reason, choose to eat a pre-prepared meal (either packaged or from a restaurant) **despite** preferring to eat something homemade.

0 1 2 3 4 5 6 7 8 9 10+

[PART 3: COOKING COMPETENCE (KNOWLEDGE AND SKILLS)]

1. What is the “claw technique” regarding safe knife usage?
   1. One hand holds the food item in the air above the cutting surface and turns the food against the knife, which is held in the other hand, to make the work more efficient (e.g., peeling an apple)
   2. The guiding hand holds pressure on top of the food item to keep it from sliding while the cut is made into the food parallel or at an angle to the work surface (e.g., slicing a bagel in half)
   3. The dominant hand grips the knife handle overhand, with the knife held vertically, for controlled and powerful strokes (e.g., de-boning meat and other fabrication techniques)
   4. ***The guiding hand’s fingertips are tucked under slightly so that the knife rests directly against the knuckles (e.g., dicing onions)***
2. Which of the following is the correct ranking of fats from lowest to highest smoke point?
   1. Extra-virgin olive oil, canola oil, whole butter
   2. Extra-virgin olive oil, whole butter, canola oil
   3. ***Whole butter, extra-virgin olive oil, canola oil***
   4. Canola oil, whole butter, extra-virgin olive oil
3. *Mirepoix, soffrito,* and the “Chinese Trinity” are all examples of what?
   1. Regional specialty dishes
   2. ***Aromatic combinations***
   3. Spice blends
   4. Condiments
4. As a general rule, from which parts of the plant are spices and herbs made?
   1. Herbs: ______________________ ***(leaves)***
   2. Spices: ______________________ ***(everything else: bark, roots, flowers, seeds)***
5. What is a roux primarily used for?
   1. To make the surface of the food item shiny
   2. To add flavor
   3. ***To thicken***
   4. To stabilize otherwise immiscible substances
6. List the 5 Mother sauces: _________, _________, _________, _________, _________

***(Béchamel, Espagnole, Velouté, Hollandaise, and Tomate)***

1. List the 5 basic tastes: _________, _________, _________, _________, _________

***(Sweet, sour, salty, bitter, and umami)***

1. What is the Maillard reaction?
   1. The reaction undergone by sugars and starches when exposed to heat that results in brown color and deep flavor
   2. ***The reaction undergone by reducing sugars and amino acids when exposed to heat that results in brown color and deep flavor***
   3. The reaction undergone by proteins when exposed to heat, acid, or salt that results in denaturation and the release of water
   4. The reaction undergone by the connective tissue in meat when exposed to heat that results in the breakdown of collagen and the formation of gelatin
2. What is the correct ordering of steps for making a basic pan sauce?
   1. ***Develop fond, add shallots, deglaze with wine, add stock, add butter***
   2. Add butter, develop fond, deglaze with wine, add shallots, add stock
   3. Add butter, add shallots, deglaze with wine, add stock, develop fond
   4. Develop fond, deglaze with wine, add butter, add shallots, add stock
3. What is the purpose of cooking with alcohol?
   1. Add flavor
   2. Incorporate fond
   3. Promote the Maillard reaction/caramelization
   4. ***All of the above***

[PART 4: COUNSELING PATIENTS ON HEALTHY LIFESTYLES]

Please rate your agreement with each of the following statements from *0-10*, where

*0* = “do not agree at all,” and

*10* = “completely agree”:

1. I have the **medical** **knowledge** necessary to effectively counsel patients on how to practice a healthy lifestyle.

0 1 2 3 4 5 6 7 8 9 10

1. I have the **nutritional** **knowledge** necessary to effectively counsel patients on how to practice a healthy lifestyle.

0 1 2 3 4 5 6 7 8 9 10

1. I have the **culinary** **knowledge** necessary to effectively counsel patients on how to practice a healthy lifestyle.

0 1 2 3 4 5 6 7 8 9 10

1. I am **prepared** to effectively counsel patients on how to practice a healthy lifestyle.

0 1 2 3 4 5 6 7 8 9 10

1. I am **motivated** to effectively counsel patients on how to practice a healthy lifestyle. 0 1 2 3 4 5 6 7 8 9 10
2. I am **excited** to effectively counsel patients on how to practice a healthy lifestyle.

0 1 2 3 4 5 6 7 8 9 10

**TIME 2: IMMEDIATELY POST-INTERVENTION SURVEY** (30 QUESTIONS) — completed via SurveyMonkey link. Phrases in bold or italics were formatted as such in SurveyMonkey. Each section was shown on a different page of the survey; participants did not see the title for any of the parts. For questions with correct answers, those correct answers are indicated in ***bold italics***. Note that for questions 6 and 7 in Part 3, each correct response was worth 0.2 points. For instance, if a participant named 2 of the 5 basic tastes correctly, they received a score of 0.4 for question 7.

[PART 1: ATTITUDES]

Please rate your agreement with each of the following statements from *0-10*, where

*0* = “do not agree at all,” and

*10* = “completely agree”:

1. I have the **medical** **knowledge** necessary to practice a healthy lifestyle.

0 1 2 3 4 5 6 7 8 9 10

1. I have the **nutritional** **knowledge** necessary to practice a healthy lifestyle.

0 1 2 3 4 5 6 7 8 9 10

1. I have the **culinary theory/knowledge** necessary to practice a healthy lifestyle.

0 1 2 3 4 5 6 7 8 9 10

1. I have the **culinary technique/skills** necessary to practice a healthy lifestyle.

0 1 2 3 4 5 6 7 8 9 10

1. I have the **motivation** necessary to practice a healthy lifestyle.

0 1 2 3 4 5 6 7 8 9 10

1. I can use culinary knowledge and skills to positively impact my **health**.

0 1 2 3 4 5 6 7 8 9 10

1. I can use culinary knowledge and skills to positively impact my **wellness**.

0 1 2 3 4 5 6 7 8 9 10

[PART 2: BEHAVIOR]

Please estimate the number of times *per week* you engage in the following behaviors:

1. Eat meals from a **restaurant** (dine-in, delivery, and takeout).

0 1 2 3 4 5 6 7 8 9 10+

1. Eat **pre-prepared meals** NOT from a restaurant (e.g., supermarket deli, frozen, or microwavable meals, etc.).

0 1 2 3 4 5 6 7 8 9 10+

1. Make a homemade **breakfast**.

0 1 2 3 4 5 6 7 8 9 10+

1. Make a homemade **lunch**.

0 1 2 3 4 5 6 7 8 9 10+

1. Make a homemade **dinner**.

0 1 2 3 4 5 6 7 8 9 10+

1. Eat **leftovers**/previously prepared homemade meals.

0 1 2 3 4 5 6 7 8 9 10+

1. For whatever reason, choose to eat a pre-prepared meal (either packaged or from a restaurant) **despite** preferring to eat something homemade.

0 1 2 3 4 5 6 7 8 9 10+

[PART 3: COOKING COMPETENCE (KNOWLEDGE AND SKILLS)]

1. What is the “claw technique” regarding safe knife usage?
   1. One hand holds the food item in the air above the cutting surface and turns the food against the knife, which is held in the other hand, to make the work more efficient (e.g., peeling an apple)
   2. The guiding hand holds pressure on top of the food item to keep it from sliding while the cut is made into the food parallel or at an angle to the work surface (e.g., slicing a bagel in half)
   3. The dominant hand grips the knife handle overhand, with the knife held vertically, for controlled and powerful strokes (e.g., de-boning meat and other fabrication techniques)
   4. ***The guiding hand’s fingertips are tucked under slightly so that the knife rests directly against the knuckles (e.g., dicing onions)***
2. Which of the following is the correct ranking of fats from lowest to highest smoke point?
   1. Extra-virgin olive oil, canola oil, whole butter
   2. Extra-virgin olive oil, whole butter, canola oil
   3. ***Whole butter, extra-virgin olive oil, canola oil***
   4. Canola oil, whole butter, extra-virgin olive oil
3. *Mirepoix, soffrito,* and the “Chinese Trinity” are all examples of what?
   1. Regional specialty dishes
   2. ***Aromatic combinations***
   3. Spice blends
   4. Condiments
4. As a general rule, from which parts of the plant are spices and herbs made?
   1. Herbs: ______________________ ***(leaves)***
   2. Spices: ______________________ ***(everything else: bark, roots, flowers, seeds)***
5. What is a roux used for?
   1. To make the surface of the food item shiny
   2. To add flavor
   3. ***To thicken***
   4. To stabilize otherwise immiscible substances
6. List the 5 Mother sauces: _________, _________, _________, _________, _________

***(Béchamel, Espagnole, Velouté, Hollandaise, and Tomate)***

1. List the 5 basic tastes: _________, _________, _________, _________, _________

***(Sweet, sour, salty, bitter, and umami)***

1. What is the Maillard reaction?
   1. The reaction undergone by sugars and starches when exposed to heat that results in brown color and deep flavor
   2. ***The reaction undergone by reducing sugars and amino acids when exposed to heat that results in brown color and deep flavor***
   3. The reaction undergone by proteins when exposed to heat, acid, or salt that results in denaturation and the release of water
   4. The reaction undergone by the connective tissue in meat when exposed to heat that results in the breakdown of collagen and the formation of gelatin
2. What is the correct ordering of steps for making a basic pan sauce?
   1. ***Develop fond, add shallots, deglaze with wine, add stock, add butter***
   2. Add butter, develop fond, deglaze with wine, add shallots, add stock
   3. Add butter, add shallots, deglaze with wine, add stock, develop fond
   4. Develop fond, deglaze with wine, add butter, add shallots, add stock
3. What is the purpose of cooking with alcohol?
   1. Add flavor
   2. Incorporate fond
   3. Promote the Maillard reaction/caramelization
   4. ***All of the above***

[PART 4: COUNSELING PATIENTS ON HEALTHY LIFESTYLES]

Please rate your agreement with each of the following statements from *0-10*, where

*0* = “do not agree at all,” and

*10* = “completely agree”:

1. I have the **medical** **knowledge** necessary to effectively counsel patients on how to practice a healthy lifestyle.

0 1 2 3 4 5 6 7 8 9 10

1. I have the **nutritional** **knowledge** necessary to effectively counsel patients on how to practice a healthy lifestyle.

0 1 2 3 4 5 6 7 8 9 10

1. I have the **culinary** **knowledge** necessary to effectively counsel patients on how to practice a healthy lifestyle.

0 1 2 3 4 5 6 7 8 9 10

1. I am **prepared** to effectively counsel patients on how to practice a healthy lifestyle.

0 1 2 3 4 5 6 7 8 9 10

1. I am **motivated** to effectively counsel patients on how to practice a healthy lifestyle. 0 1 2 3 4 5 6 7 8 9 10
2. I am **excited** to effectively counsel patients on how to practice a healthy lifestyle.

0 1 2 3 4 5 6 7 8 9 10

**TIME 3: 8-WEEKS POST-INTERVENTION SURVEY** (30 QUESTIONS) — completed via SurveyMonkey link. Phrases in bold or italics were formatted as such in SurveyMonkey. Each section was shown on a different page of the survey; participants did not see the title for any of the parts. For questions with correct answers, those correct answers are indicated in ***bold italics***. Note that for questions 6 and 7 in Part 3, each correct response was worth 0.2 points. For instance, if a participant named 2 of the 5 basic tastes correctly, they received a score of 0.4 for question 7.

[PART 1: ATTITUDES]

Please rate your agreement with each of the following statements from *0-10*, where

*0* = “do not agree at all,” and

*10* = “completely agree”:

1. I have the **medical** **knowledge** necessary to practice a healthy lifestyle.

0 1 2 3 4 5 6 7 8 9 10

1. I have the **nutritional** **knowledge** necessary to practice a healthy lifestyle.

0 1 2 3 4 5 6 7 8 9 10

1. I have the **culinary theory/knowledge** necessary to practice a healthy lifestyle.

0 1 2 3 4 5 6 7 8 9 10

1. I have the **culinary technique/skills** necessary to practice a healthy lifestyle.

0 1 2 3 4 5 6 7 8 9 10

1. I have the **motivation** necessary to practice a healthy lifestyle.

0 1 2 3 4 5 6 7 8 9 10

1. I can use culinary knowledge and skills to positively impact my **health**.

0 1 2 3 4 5 6 7 8 9 10

1. I can use culinary knowledge and skills to positively impact my **wellness**.

0 1 2 3 4 5 6 7 8 9 10

[PART 2: BEHAVIOR]

Please estimate the number of times *per week* you engage in the following behaviors:

1. Eat meals from a **restaurant** (dine-in, delivery, and takeout).

0 1 2 3 4 5 6 7 8 9 10+

1. Eat **pre-prepared meals** NOT from a restaurant (e.g., supermarket deli, frozen, or microwavable meals, etc.).

0 1 2 3 4 5 6 7 8 9 10+

1. Make a homemade **breakfast**.

0 1 2 3 4 5 6 7 8 9 10+

1. Make a homemade **lunch**.

0 1 2 3 4 5 6 7 8 9 10+

1. Make a homemade **dinner**.

0 1 2 3 4 5 6 7 8 9 10+

1. Eat **leftovers**/previously prepared homemade meals.

0 1 2 3 4 5 6 7 8 9 10+

1. For whatever reason, choose to eat a pre-prepared meal (either packaged or from a restaurant) **despite** preferring to eat something homemade.

0 1 2 3 4 5 6 7 8 9 10+

[PART 3: COOKING COMPETENCE (KNOWLEDGE AND SKILLS)]

1. What is the “claw technique” regarding safe knife usage?
   1. One hand holds the food item in the air above the cutting surface and turns the food against the knife, which is held in the other hand, to make the work more efficient (e.g., peeling an apple)
   2. The guiding hand holds pressure on top of the food item to keep it from sliding while the cut is made into the food parallel or at an angle to the work surface (e.g., slicing a bagel in half)
   3. The dominant hand grips the knife handle overhand, with the knife held vertically, for controlled and powerful strokes (e.g., de-boning meat and other fabrication techniques)
   4. ***The guiding hand’s fingertips are tucked under slightly so that the knife rests directly against the knuckles (e.g., dicing onions)***
2. Which of the following is the correct ranking of fats from lowest to highest smoke point?
   1. Extra-virgin olive oil, canola oil, whole butter
   2. Extra-virgin olive oil, whole butter, canola oil
   3. ***Whole butter, extra-virgin olive oil, canola oil***
   4. Canola oil, whole butter, extra-virgin olive oil
3. *Mirepoix, soffrito,* and the “Chinese Trinity” are all examples of what?
   1. Regional specialty dishes
   2. ***Aromatic combinations***
   3. Spice blends
   4. Condiments
4. As a general rule, from which parts of the plant are spices and herbs made?
   1. Herbs: ______________________ ***(leaves)***
   2. Spices: ______________________ ***(everything else: bark, roots, flowers, seeds)***
5. What is a roux used for?
   1. To make the surface of the food item shiny
   2. To add flavor
   3. ***To thicken***
   4. To stabilize otherwise immiscible substances
6. List the 5 Mother sauces: _________, _________, _________, _________, _________

***(Béchamel, Espagnole, Velouté, Hollandaise, and Tomate)***

1. List the 5 basic tastes: _________, _________, _________, _________, _________

***(Sweet, sour, salty, bitter, and umami)***

1. What is the Maillard reaction?
   1. The reaction undergone by sugars and starches when exposed to heat that results in brown color and deep flavor
   2. ***The reaction undergone by reducing sugars and amino acids when exposed to heat that results in brown color and deep flavor***
   3. The reaction undergone by proteins when exposed to heat, acid, or salt that results in denaturation and the release of water
   4. The reaction undergone by the connective tissue in meat when exposed to heat that results in the breakdown of collagen and the formation of gelatin
2. What is the correct ordering of steps for making a basic pan sauce?
   1. ***Develop fond, add shallots, deglaze with wine, add stock, add butter***
   2. Add butter, develop fond, deglaze with wine, add shallots, add stock
   3. Add butter, add shallots, deglaze with wine, add stock, develop fond
   4. Develop fond, deglaze with wine, add butter, add shallots, add stock
3. What is the purpose of cooking with alcohol?
   1. Add flavor
   2. Incorporate fond
   3. Promote the Maillard reaction/caramelization
   4. ***All of the above***

[PART 4: COUNSELING PATIENTS ON HEALTHY LIFESTYLES]

Please rate your agreement with each of the following statements from *0-10*, where

*0* = “do not agree at all,” and

*10* = “completely agree”:

1. I have the **medical** **knowledge** necessary to effectively counsel patients on how to practice a healthy lifestyle.

0 1 2 3 4 5 6 7 8 9 10

1. I have the **nutritional** **knowledge** necessary to effectively counsel patients on how to practice a healthy lifestyle.

0 1 2 3 4 5 6 7 8 9 10

1. I have the **culinary** **knowledge** necessary to effectively counsel patients on how to practice a healthy lifestyle.

0 1 2 3 4 5 6 7 8 9 10

1. I am **prepared** to effectively counsel patients on how to practice a healthy lifestyle.

0 1 2 3 4 5 6 7 8 9 10

1. I am **motivated** to effectively counsel patients on how to practice a healthy lifestyle. 0 1 2 3 4 5 6 7 8 9 10
2. I am **excited** to effectively counsel patients on how to practice a healthy lifestyle.

0 1 2 3 4 5 6 7 8 9 10
